# Cerebral spinal fluid biomarker profiles in CNS infection associated with HSV and VZV mimic patterns in Alzheimer’s disease

**DOI:** 10.1186/s40035-020-00227-w

**Published:** 2021-01-04

**Authors:** Makiko Shinomoto, Takashi Kasai, Harutsugu Tatebe, Fukiko Kitani-Morii, Takuma Ohmichi, Yuzo Fujino, David Allsop, Toshiki Mizuno, Takahiko Tokuda

**Affiliations:** 1 Department of Neurology, Kyoto Prefectural University of Medicine, Kyoto, 602-0841 Japan; 2 Department of Functional Brain Imaging Research, National Institutes for Quantum and Radiological Science and Technology, Chiba, 263-8555 Japan; 3 Division of Biomedical and Life Sciences, Faculty of Health and Medicine, Lancaster University, Lancaster, LA1 4YQ UK

Alzheimer’s disease (AD) is the most common cause of dementia. Although AD was initially considered to be a cell autonomous neurodegenerative disorder, marked neuroinflammation has been observed in brains of AD patients. Genetic and molecular biological findings have suggested the central nervous system (CNS) inflammatory processes to be involved in the etiopathogenesis of AD, in which the activated microglia play a key role. This has also been supported by epidemiological observation that CNS infections are associated with the development of AD, and the relationship between herpes simplex virus (HSV)-1 and AD has been particularly well investigated [1]. For example, the presence of anti-HSV antibody is associated with an elevated risk of AD [2]. Anti-herpetic medication is associated with a reduced risk of dementia in a population-based study [1]. Similar results have also been observed in varicella zoster virus (VZV) infections [3]. In this study, we enrolled 9 patients with HSV infection of the CNS, 8 patients with herpes zoster complicated by CNS involvement, and 18 age-matched control patients presenting with neither CNS infection nor dementia, and measured cerebral spinal fluid (CSF) levels of Aβ_1–42_, Aβ_1–40_, total-tau (t-tau), and phosphorylated tau at threonine 181 (p-tau) as the AD signature; neurofilament light chain (NfL) and phosphorylated neurofilament heavy chain (p-NfH) as indicators of axonal injury; soluble triggering receptor expressed on myeloid cells 2 (sTREM2) as a potential biomarker for microglial activity; and glial fibrillary acidic protein (GFAP) as a biomarker for astrocytic damage. We also measured serum levels of NfL as a blood-based biomarker for axonal injury. Detailed methods are provided in Supplementary Methods. There was no significant difference in age or sex among the HSV, VZV, and control groups (Table S1). The raw data on biomarkers are presented in Table S2.

We found that the levels of CSF Aβ_1–42_, Aβ_1–40_, and the Aβ_1–42_/Aβ_1–40_ ratio were significantly lower in the HSV + VZV combined group (HSV/VZV) compared with the control group (*p* = 0.01836, 0.0380, and 0.0262, respectively) (Fig. 1a–c). The CSF t-tau, p-tau, sTREM2, and GFAP levels were significantly elevated in the HSV/VZV group compared with the control group (*p* = 0.0043, 0.0007, 0.0030, and 0.0139, respectively) (Fig. 1d, e, i, and j). These results correspond to previous reports showing significantly decreased Aβ_1–42_, increased t-tau, and increased p-tau in CSF of patients with HSV encephalitis [4, 5]. The CSF p-tau/t-tau, CSF NfL, CSF p-NfH and serum NfL levels did not significantly differ between the HSV/VZV and control groups (Fig. 1f, g, h, and k). Comparison among the HSV, VZV and control groups showed that the elevation of CSF p-tau was significant in the VZV group while the level of CSF t-tau was elevated specifically in the HSV group (Supplementary Fig. S1). The other biomarkers showed similar trends to those in comparison between HSV/VZV and the control groups.

**Fig. 1:**
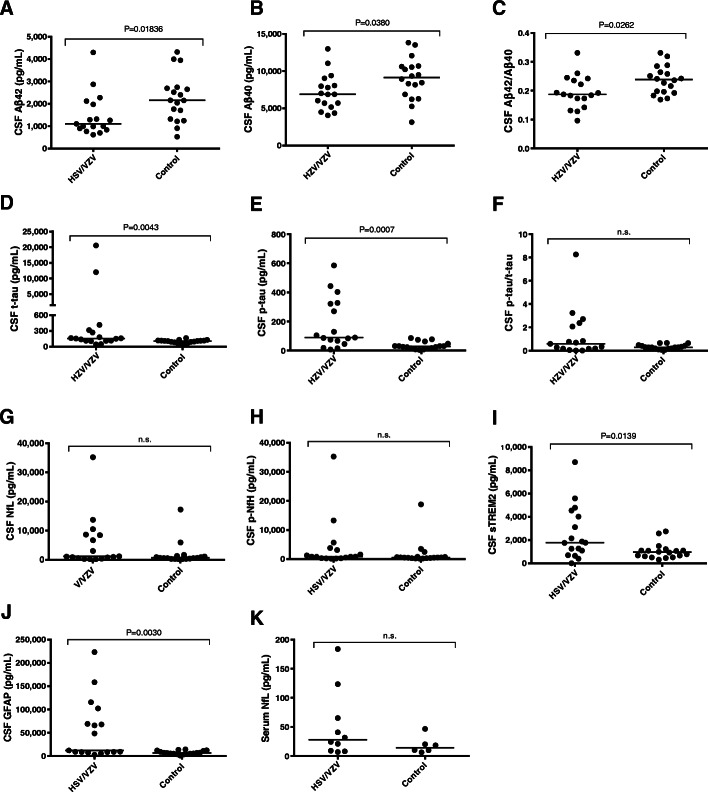
Comparisons of (**a**) CSF Aβ_1–42_, (**b**) CSF Aβ_1–40_, (**c**) CSF Aβ_1–42_/Aβ_1–40_, (**d**) CSF t-tau, (**e**) CSF p-tau, (**f**) CSF p-tau/t-tau ratio, (**g**) CSF NfL, (**h**) CSF pNfH, (**i**) CSF sTREM2, (**j**) CSF GFAP, and (**k**) serum NfL between the HSV/VZV and control groups. The CSF (HSV/VZV group: *n* = 17; control: *n* = 18) and serum (HSV/VZV group: *n* = 9; control: *n* = 6) levels of those biomarkers in each individual are shown as black dots. Bars indicate median values. n.s., not significant

Results of uni- and multivariate regression analyses between those biomarker values and clinical severity are summarized in Supplementary Table S3. The negative correlations between Glasgow coma scale and NfL in CSF and serum were significant after age adjustment (*p* = 0.014 and 0.030, respectively). Among the biomarkers, only CSF NfL was significantly correlated with the Modified Rankin Scale on discharge after age adjustment (*p* = 0.018). Supplementary Fig. S2 shows scatter plots in cases showing significant correlations on univariate analyses.

The current study has three major implications. First, to our best knowledge, this is the first report of CSF p-tau elevation in patients with CNS VZV infection. This suggests that the biomarker profile of decreased Aβ_1–42_, and increased t-tau and p-tau in CSF might be shared not only by CNS involvement of HSV infection but also by CNS VZV infection. This combination of biomarker changes, the so-called “AD signature”, has been considered to indicate the presence of AD pathology. In particular, high levels of p-tau at threonine181 have been reported to occur solely in AD and not in other neurodegenerative disorders or acute brain damage, such as acute brain infarction [6]. The CSF p-tau elevation in the HSV/VZV group might be attributed to the herpetic infection-induced APP mis-metabolism [7], as similarly seen in the case of AD. In addition, the fact that the biomarker profile in AD patients mimics that in patients with CNS HSV and VZV infections suggests that the latter may be a confounding factor in the CSF biomarker-based diagnosis.

Second, the elevations of CSF sTREM2 and GFAP in the HSV and VZV groups are in line with previous observations [8]. These trends are also consistent with the reported biomarker changes in patients with AD [9, 10].

Third, only the NfL levels were significantly correlated with the severity and a poor outcome after age adjustment in the CSF biomarkers. This suggests that the NfL concentration in CSF obtained for the diagnostic purpose on admission is the most powerful predictive marker for the severity and prognosis in patients with herpes virus infections among the molecules tested in this study. The CSF and blood NfL levels are reported to be tightly correlated in neurological disorders. Here we found that the serum levels of NfL were strongly associated with their matching CSF levels (Supplementary Fig. S3) and consequently, the ability of serum NfL to evaluate the severity and to predict prognosis may be equivalent to those of CSF NfL.

We acknowledge that the small sample size was a major limitation of this study. Furthermore, the short follow-up period may have weakened the statistical power to detect an association between the prognosis and biomarkers. In the future, it will be necessary to conduct large-scale case-control studies and prospective observations in order to validate the clinical significance of AD-related biomarkers in patients with CNS HSV and VZV infections.

## Supplementary Information

**Additional file 1: Supplementary methods. Table S1:** Characteristics of the participants. **Table S2.** The concentrations of biomarkers in participants. **Table S3.** Regression analyses of clinical status and biomarkers. **Figure S1.** Comparison of biomarker concentrations among the HSV, VZV, and control groups (A: CSF Aβ_1–42_, B: CSF Aβ_1–40_, C: CSF Aβ_1–42/1–40_ ratio, D: CSF t-tau, E: CSF p-tau, F: CSF p-tau/t-tau ratio, G: CSF NfL, H: CSF pNfH, I: CSF sTREM2, J: CSF GFAP, and K: serum NfL). **Figure S2.** Scatter plots of the biomarkers vs the lowest score of GCS during hospitalization (A, B, C, D) as well as the biomarkers *vs* mRS score at discharge (E, F, G, H, I, J, K) in the HSV/VZV group. **Figure S3.** Correlations between CSF and serum NfL levels. There was a strong positive correlation between CSF and serum NfL levels by Spearman’s rank correlation test (*p* < 0.0001).

## Data Availability

All data generated or analyzed during this study are included in this published article and its supplementary information files.

## Abbreviations

CNS: Central nervous system
AD: Alzheimer’s disease
HSV: Herpes simplex virus
VZV: Varicella zoster virus
Aβ: Amyloid β
t-tau: Total tau
p-tau: Tau phosphorylated at threonine 181
NfL: Neurofilament light chain
p-NfH: Phosphorylated neurofilament heavy chain
GFAP: Glial fibrillary acidic protein
sTREM2: Soluble triggering receptor expressed on myeloid cells 2
CSF: Cerebrospinal fluid

## References

[1] Itzhaki RF. Corroboration of a major role for herpes simplex virus type 1 in Alzheimer’s disease. Front Aging Neurosci. 2018;10:324. 10.3389/fnagi.2018.00324 PMC6202583 30405395

[2] Lovheim H, Gilthorpe J, Johansson A, Eriksson S, Hallmans G, Elgh F (2015). Herpes simplex infection and the risk of Alzheimer's disease: a nested case-control study. Alzheimers Dement.

[3] Chen VC, Wu SI, Huang KY, Yang YH, Kuo TY, Liang HY, et al. Herpes zoster and dementia: a nationwide population-based cohort study. J Clin Psychiatry. 2018;79(1):16m11312. 10.4088/JCP.16m11312 29244265

[4] Krut JJ, Zetterberg H, Blennow K, Cinque P, Hagberg L, Price RW (2013). Cerebrospinal fluid Alzheimer's biomarker profiles in CNS infections. J Neurol.

[5] Di Stefano A, Alcantarini C, Atzori C, Lipani F, Imperiale D, Burdino E (2020). Cerebrospinal fluid biomarkers in patients with central nervous system infections: a retrospective study. CNS Spectrums.

[6] Blennow K, Zetterberg H (2018). Biomarkers for Alzheimer's disease: current status and prospects for the future. J Intern Med.

[7] Civitelli L, Marcocci ME, Celestino I, Piacentini R, Garaci E, Grassi C (2015). Herpes simplex virus type 1 infection in neurons leads to production and nuclear localization of APP intracellular domain (AICD): implications for Alzheimer's disease pathogenesis. J Neuro-Oncol.

[8] Grahn A, Hagberg L, Nilsson S, Blennow K, Zetterberg H, Studahl M (2013). Cerebrospinal fluid biomarkers in patients with varicella-zoster virus CNS infections. J Neurol.

[9] Suárez-Calvet M, Araque Caballero MÁ, Kleinberger G, Bateman RJ, Fagan AM, Morris JC (2016). Early changes in CSF sTREM2 in dominantly inherited Alzheimer’s disease occur after amyloid deposition and neuronal injury. Sci Transl Med.

[10] Oeckl P, Halbgebauer S, Anderl-Straub S, Steinacker P, Huss AM, Neugebauer H (2019). Glial fibrillary acidic protein in serum is increased in Alzheimer's disease and correlates with cognitive impairment. J Alzheimers Dis.

