# Supplementary material for: Cerebral spinal fluid biomarker profiles in CNS infection associated with HSV and VZV mimic patterns in Alzheimer’s disease
Source: Transl Neurodegener. 2021 Jan 4;10:2. doi: 10.1186/s40035-020-00227-w (PMC7780670; doi:10.1186/s40035-020-00227-w)
Supplement: Supplementary file 1 — Additional file 1: Supplementary methods. Table S1: Characteristics of the participants. Table S2. The concentrations of biomarkers in participants. Table S3. Regression analyses of clinical status and biomarkers. Figure S1. Comparison of biomarker concentrations among the HSV, VZV, and control groups (A: CSF Aβ1–42, B: CSF Aβ1–40, C: CSF Aβ1–42/1–40 ratio, D: CSF t-tau, E: CSF p-tau, F: CSF p-tau/t-tau ratio, G: CSF NfL, H: CSF pNfH, I: CSF sTREM2, J: CSF GFAP, and K: serum NfL). Figure S2. Scatter plots of the biomarkers vs the lowest score of GCS during hospitalization (A, B, C, D) as well as the biomarkers vs mRS score at discharge (E, F, G, H, I, J, K) in the HSV/VZV group. Figure S3. Correlations between CSF and serum NfL levels. There was a strong positive correlation between CSF and serum NfL levels by Spearman’s rank correlation test (p < 0.0001). [file 40035_2020_227_MOESM1_ESM.docx]

**Supplementary file of**

**CSF biomarker profiles in CNS infection associated with HSV and VZV mimic pattern in Alzheimer’s disease.**

Makiko Shinomoto^1^ (makiko-t@koto.kpu-m.ac.jp)

Takashi Kasai^1^ ([kasaita@koto.kpu-m.ac.jp](mailto:kasaita@koto.kpu-m.ac.jp))

Harutsugu Tatebe^2^(tatebe.harutsugu@qst.go.jp)

Fukiko Kitani-Morii ^1^(f-morii@koto.kpu-m.ac.jp)

Takuma Ohmichi^1^ ([t-omichi@koto.kpu-m.ac.jp](mailto:t-omichi@koto.kpu-m.ac.jp))

Yuzo Fujino^1^ (fujino@koto.kpu-m.ac.jp)

David Allsop^3^(d.allsop@lancaster.ac.uk)

Toshiki Mizuno^1^ (mizuno@koto.kpu-m.ac.jp)

Takahiko Tokuda^2^ (tokuda.takahiko@qst.go.jp)

^1^ Department of Neurology, Kyoto Prefectural University of Medicine, Kyoto 602-0841, Japan

^2^ Department of Functional Brain Imaging Research, National Institute of Radiological Sciences, National Institutes for Quantum and Radiological Science and Technology 263-8555, Japan

^3^ Division of Biomedical and Life Sciences, Faculty of Health and Medicine, Lancaster University, Lancaster LA1 4YQ, UK

Corresponding author:

Corresponding authors: Takashi Kasai, M.D., Ph.D. and Takahiko Tokuda, M.D., Ph.D.

Department of Neurology, Kyoto Prefectural University of Medicine, Kyoto 602-0841 for TK, Japan and Department of Functional Brain Imaging Research, National Institute of Radiological Sciences, National Institutes for Quantum and Radiological Science and Technology 263-8555, Japan for TT

Tel.: +81-75-251-5793 Fax: +81-75-211-8645

E-mail: kasaita@koto.kpu-m.ac.jp to TK, tokuda.takahiko@qst.go.jp to TT

**Supplementary methods**

***Study design, ethics statement, and subject recruitment***

All study subjects provided written informed consent before participation and the study protocols were approved by the University Ethics Committee (ERB-G-12, Kyoto Prefectural University of Medicine, Kyoto, Japan). Written informed consent from the participants was obtained when possible and, if not, from the nearest relative. Study procedures were designed and performed in accordance with the Declaration of Helsinki. Diagnosis of CNS HSV involvement was conducted in accordance with a published guideline for HSV encephalitis [1] (based on the presence of pleocytosis (≥5 μL) with neurological symptoms including headache and positive HSV/VZV PCR results in CSF or elevation of IgM to HSV/VZV in serum and/or CSF). The diagnosis was also supported by MRI abnormalities consistent with encephalitis, myelitis, or leptomeningeal gadolinium enhancement. Patients presenting with dementia before the onset of CNS infection were excluded. We also excluded patients without pleocytosis in CSF (i.e., patients presenting with only cutaneous shingles or serum HSV IgM elevation without pleocytosis in CSF were not included in the study). We enrolled 9 patients with HSV infection of the CNS (HSV group: 7 patients with HSV encephalitis, 2 patients with HSV meningitis, and 1 patient with HSV myelitis), 8 patients with herpes zoster complicated by CNS involvement (VZV group: 7 patients with meningitis, 1 patient with myelitis). Eighteen age-matched control patients presenting with neither CNS infection nor dementia were also enrolled (control group). All patients visited Kyoto Prefectural University of Medicine between 1990 and 2019 and underwent lumbar puncture for clinical reasons. CSF samples were collected in polypropylene vials from cases in the morning (from 9 a.m. to 12 a.m.) through a lumbar puncture at the L3/L4 or L4/L5 interspace. Immediately after collection, the samples were cleared by centrifugation at 400 x g for 10 min at 4°C. When blood collection was done at the same time as CSF collection, serum was separated by centrifugation for 10 min at 3,000 g after the blood was allowed to clot for 15 min at room temperature in blood collection tubes with clot activator and gel separator (Terumo, Tokyo, Japan), and transferred to polypropylene vials. Fresh samples obtained from the enrolled subjects were immediately stored at -80°C until analysis.

***Measurements of CSF and serum biomarkers***

We analyzed CSF and serum samples of the HSV and VZV groups obtained on admission for diagnostic purposes, except for case 1 of the VZV group whose CSF sample was obtained on day 4 due to an inadequate volume of CSF on admission. We measured the concentrations of Aβ_1-42_, Aβ_1-40,_ and t-tau in CSF with reagents from a single lot using the Simoa Human Neurology 3-Plex A assay and the concentrations of NfL in CSF and serum, p-NfH, and GFAP in CSF with reagents from a single lot using the Simoa NF-light assay, Simoa pNF-heavy assay, and Simoa GFAP assay, respectively. All measurements were performed on an HD-1 Simoa analyzer according to the protocol provided by the manufacturer (Quanterix, Lexington, MA, USA). The CSF levels of p-tau181 were analyzed using the originally developed p-tau181 assay on the Simoa platform, reported elsewhere [2]. The CSF sTREM2 levels were quantified by ELISA using a RayBio Human TREM-2 ELISA Kit (RayBiotech, Norcross, GA, USA) in accordance with the manufacturer’s instructions and a previous report [3].

All samples were analyzed in duplicate on one occasion. All cases and controls were evenly distributed on examination.

***Statistics***

The level of significance was set at P<0.05. A comparison between two independent groups was performed using the Mann-Whitney U test, and a comparison among three independent groups was performed using the Kruskal-Wallis test and Dunn’s multiple comparison procedure. The Chi-square test was used to evaluate the significance of categorical variables. The uni- and multivariate regression analyses were performed using Spearman’s rank correlation coefficient test and ordinal logistic regression model, respectively. Comparison among the groups was performed using GraphPad Prism software (version 8.4.2, GraphPad Software, San Diego, USA). Regression analyses were performed using SPSS for Windows version 23 software (IBM Japan Ltd., Tokyo, Japan).

**References**

1. Tunkel AR, Glaser CA, Bloch KC, Sejvar JJ, Marra CM, Roos KL, Hartman BJ, Kaplan SL, Scheld WM, Whitley RJ: **The management of encephalitis: clinical practice guidelines by the Infectious Diseases Society of America.** *Clin Infect Dis* 2008, **47:**303-327.

2. Tatebe H, Kasai T, Ohmichi T, Kishi Y, Kakeya T, Waragai M, Kondo M, Allsop D, Tokuda T: **Quantification of plasma phosphorylated tau to use as a biomarker for brain Alzheimer pathology: pilot case-control studies including patients with Alzheimer's disease and down syndrome.** *Mol Neurodegener* 2017, **12:**63.

3. Ohara T, Hata J, Tanaka M, Honda T, Yamakage H, Yoshida D, Inoue T, Hirakawa Y, Kusakabe T, Shibata M, et al: **Serum Soluble Triggering Receptor Expressed on Myeloid Cells 2 as a Biomarker for Incident Dementia: The Hisayama Study.** *Ann Neurol* 2019, **85:**47-58.

**Supplementary Table S1:** Characteristics of the participants.

| Case | Sex | Age | Diagnosis | CSF PCR | CSF HSV IgM | Serum HSV IgM | CSF cell count (/μL) | CSF protein (mg/dL) | CSF collection date (days after admission) | Treatment | degree of MRI finding | GCS | mRS | Cognitive sequelae at discharge | Level of consciousness |
| --- | --- | --- | --- | --- | --- | --- | --- | --- | --- | --- | --- | --- | --- | --- | --- |
| 1^*^ | M | 52 | Encephalitis | (-) | (-) | (+) | 28 | 60 | 17^#^ | Aci | 1 | 13 | 2 | Amnestic aphasia | Disorientation as to time with aphasia |
| 2^*^ | M | 45 | Encephalitis | (-) | (-) | (+) | 99 | 62 | 7^#^ | Aci+CS | 4 | 15 | 0 | None | Disorientation as to time |
| 3^*^ | M | 56 | Encephalitis | (-) | (-) | (+) | 26 | 77 | 0^$^ | Aci+AE | 2 | 14 | 3 | Memory impairment | Disorientation as to time, place, and self, with convulsion |
| 4^*^ | F | 22 | Encephalitis | (+) | (+) | (+) | 62 | 37 | 4 | Aci+CS | 3 | 11 | 2 | None | Disorientation as to time, place, and self |
| 5^*^ | F | 66 | Encephalitis | (+) | N/A | N/A | 24 | 83 | 13^#^ | Aci+AraA+CS+AE | 4 | 13 | 3 | Amnestic aphasia | Disorientation as to time with aphasia |
| 6^*^ | F | 56 | Myelitis | (-) | N/A | (+) | 7 | 30 | 9^#^ | Aci+CS | N/A (Th2-4)* | 15 | 2 | None | Alert and oriented |
| 7 | F | 33 | Meningitis | N/A | (+) | (-) | 364 | 155 | 0^$^ | Aci | 1 | 15 | 0 | None | Alert and oriented |
| 8 | F | 47 | Meningitis | N/A | N/A | (+) | 38 | 59 | 9^#^ | Aci | 1 | 15 | 0 | None | Alert and oriented |
| 9^*^ | M | 83 | Encephalitis | (+) | N/A | (+) | 249 | 271 | 16^#^ | Aci | 2 | 3 | 4 | Memory impairment | Coma with convulsion |

A:

^#^Note: In cases 1, 2, 5, 6, 8, and 9, we used the second sample collected after admission, because the first CSF sample was not preserved.

^$^Note: In cases 3 and 7, the treatment shown above did not start at the time of CSF collection.

Abbreviations in the treatment column: Aci: acyclovir, CS: corticosteroid, AE: anti-epileptic medications, AraA: vidarabine

The degree of brain MRI involvement in the HSV/VZV group was categorized into the following five grades: Grade 0: no apparent abnormality on MRI Grade 1: leptomeningeal gadolinium enhancement on MRI without apparent abnormality on T2WI or FLAIR; Grade 3: unilateral imaging abnormality on T2WI or FLAIR spreading from the medial to lateral temporal lobe; Grade 4: unilateral imaging abnormality on T2WI or FLAIR spreading from the temporal lobe to other lobes except for the temporal lobe; Grade 5: bilateral imaging abnormality on T2WI or FLAIR. Note: case 6 was not categorized because of the lack of brain involvement (spinal cord involvement at Th 2-4). GCS showed the lowest score during hospitalization. The scores of mRS are those at discharge. N/A: not available.

B:

| Case | Sex | Age | Diagnosis | CSF PCR | CSF VZV IgM | Serum VZV IgM | CSF cell count (/μL) | CSF protein (mg/dL) | CSF collection date (days after admission) | Treatment | Degree of MRI finding | GCS | mRS | Cognitive sequelae at discharge | Level of consciousness |
| --- | --- | --- | --- | --- | --- | --- | --- | --- | --- | --- | --- | --- | --- | --- | --- |
| 1 | M | 32 | Myelitis | (-) | (-) | (+) | 3^#^ | 28 | 14 | Aci+CS | Medulla oblongata to C2 | 15 | 2 | None | Alert and oriented |
| 2^*^ | F | 84 | Meningitis | (-) | (-) | (+) | 99 | 129 | 0^$^ | Aci | 0 | 14 | 3 | None | Disorientation as to time, place, and self; |
| 3 | M | 21 | Meningitis | (+) | N/A | N/A | 91 | 54 | 0^$^ | Aci | 1 | 15 | 0 | None | Alert and oriented |
| 4^*^ | F | 27 | Meningitis | (+) | N/A | N/A | 303 | 80 | 4 | Aci | 1 | 14 | 0 | None | Alert and oriented |
| 5 | M | 70 | Meningitis | (+) | N/A | N/A | 95 | 206 | 8 | Aci | 0 | 15 | 1 | None | Alert and oriented |
| 6^*^ | F | 15 | Meningitis | (+) | N/A | N/A | 1312 | 114 | 0^$^ | Aci | 0 | 15 | 0 | None | Alert and oriented |
| 7 | M | 55 | Encephalitis | (+) | N/A | N/A | 660 | 193 | 0^$^ | Aci+AE | 0 | 6 | 0 | None | Disorientation as to time, place, and self with convulsion |
| 8 | F | 71 | Meningitis | N/A | N/A | N/A | 5 | 32 | 0^$^ | Aci | 0 | 15 | 0 | None | Alert and oriented |

^#^Note: Case 1 showed CSF pleocytosis (13/μL) at the first lumbar puncture. Because we ran out of the initial CSF sample, we used the sample collected 3 days after the first examination.

^$^Note: In cases 2, 3, 6, and 7, the treatment shown above did not start at the time of CSF collection.

Abbreviations in the treatment column: Aci: acyclovir, CS: corticosteroid, AE: anti-epileptic medications

The degree of brain MRI involvement in the HSV/VZV group was categorized into the following five grades: Grade 0: no apparent abnormality on MRI Grade 1: leptomeningeal gadolinium enhancement on MRI without apparent abnormality on T2WI or FLAIR; Grade 3: unilateral imaging abnormality on T2WI or FLAIR spreading from the medial to lateral temporal lobe; Grade 4: unilateral imaging abnormality on T2WI or FLAIR spreading from the temporal lobe to other lobes except for the temporal lobe; Grade 5: bilateral imaging abnormality on T2WI or FLAIR. Note: case 1 was not categorized because of the brain stem and upper cervical involvment. GCS showed the lowest score during hospitalization. The scores of mRS are those at discharge. N/A: not available.

C:

| Case | Sex | Age | Diagnosis or symptom | CSF cell count (/μL) | CSF protein (mg/dL) | CSF PCR of HSV and VZV | CSF HSV and VZV IgM | Serum HSV and VZV IgM |
| --- | --- | --- | --- | --- | --- | --- | --- | --- |
| 1^*^ | F | 42 | Influenza | ~~6~~ 2 | 38 | N/A | N/A | N/A |
| 2 | F | 25 | Fever elevation | ~~12~~ 1 | 25 | N/A | N/A | N/A |
| 3 | M | 29 | Fever elevation | ~~36~~ 4 | 30 | N/A | N/A | N/A |
| 4^*^ | F | 72 | Crowned dens syndrome | ~~6~~ 1 | 39 | N/A | (-) | (-) |
| 5 | F | 36 | Fever elevation | ~~21~~ 2 | 32 | N/A | (-) | (-) |
| 6^*^ | M | 71 | Cervical spondylosis | ~~6~~ 2 | 49 | N/A | N/A | N/A |
| 7 | F | 73 | Neuralgic amyotrophy | ~~9~~ 3 | 39 | N/A | N/A | N/A |
| 8 | M | 50 | Cervical spondylosis | ~~12~~ 4 | 187 | N/A | N/A | N/A |
| 9 | M | 66 | Fatigue | ~~3~~ 1 | 35 | N/A | N/A | N/A |
| 10 | M | 77 | Oculomotor paresis | ~~3~~ 1 | 42 | N/A | N/A | N/A |
| 11^*^ | M | 67 | Dysphagia | ~~3~~ 1 | 56 | N/A | N/A | N/A |
| 12 | M | 44 | Viral myositis | ~~3~~ 1 | 36 | N/A | N/A | N/A |
| 13 | F | 63 | Transient global amnesia | 0 | 19 | N/A | N/A | N/A |
| 14^*^ | M | 44 | Psychophysiologic disorder | ~~9~~ 3 | 28 | N/A | (-) | (-) |
| 15^*^ | M | 37 | Peroneal nerve paralysis | ~~6~~ 2 | 33 | N/A | N/A | N/A |
| 16 | F | 49 | Fever elevation | ~~3~~ 1 | 24 | N/A | N/A | N/A |
| 17 | M | 59 | Brachial plexus neuropathy | ~~3~~ 0 | 36 | N/A | N/A | N/A |
| 18 | F | 24 | Guillain-Barré syndrome | ~~24~~ 3 | 29 | N/A | N/A | N/A |

Clinical characteristics of the HSV (A), VZV (B), and control (C) groups are shown.

Cases who also measured serum NfL were indicated by asterisks.

**Supplementary Table S2:** The concentration of biomarkers in participants.

| Case | CSF Aβ42  (pg/mL) | CSF Aβ40  (pg/mL) | CSF t-tau  (pg/mL) | CSF p-tau  (pg/mL) | CSF NfL  (pg/mL) | CSF GFAP  (pg/mL) | CSF p-NfH  (pg/mL) | CSF sTREM-2  (pg/mL) | Serum NfL (pg/mL) |
| --- | --- | --- | --- | --- | --- | --- | --- | --- | --- |
| HSV group | |  |  |  |  |  |  |  |  |
| 1 | 703.97 | 4068.86 | 269.09 | 89.73 | 8746.84 | 158443.99 | 5689.09 | 4535.06 | 65.17 |
| 2 | 987.10 | 5721.85 | 114.64 | 67.48 | 680.11 | 8916.01 | 259.53 | 4777.45 | 8.01 |
| 3 | 619.48 | 4365.47 | 315.13 | 16.00 | 1029.04 | 5347.40 | 825.44 | 392.17 | 24.20 |
| 4 | 1002.62 | 5185.24 | 20540.65 | 270.24 | 6832.21 | 115439.96 | 1288.69 | 695.00 | 40.79 |
| 5 | 1319.10 | 6881.71 | 12016.41 | 129.97 | 13800.64 | 65760.89 | 3819.92 | 8696.64 | 31.69 |
| 6 | 1977.94 | 8067.88 | 112.39 | 18.71 | 3130.32 | 69128.69 | 3104.44 | 1258.08 | 20.96 |
| 7 | 1105.29 | 6040.32 | 138.47 | 327.99 | 493.19 | 7435.83 | 311.92 | 1269.19 | N/A |
| 8 | 2872.99 | 11055.36 | 151.68 | 104.81 | 1076.11 | 9225.18 | 795.62 | 1094.11 | N/A |
| 9 | 997.51 | 7812.42 | 413.83 | 75.27 | 35303.47 | 67711.08 | 35246.34 | 3113.54 | 183.76 |
| VZV group | |  |  |  |  |  |  |  |  |
| 1 | 841.25 | 4501.62 | 42.96 | 7.46 | 397.24 | 2714.43 | 267.72 | 0* | N/A |
| 2 | 769.53 | 7978.25 | 163.19 | 442.69 | 10600.88 | 223196.79 | 1581.11 | 5577.59 | 123.42 |
| 3 | 1265.23 | 5711.28 | 48.73 | 402.73 | 309.83 | 8484.92 | 139.96 | 646.70 | N/A |
| 4 | 4297.00 | 12992.93 | 180.55 | 584.67 | 1176.14 | 9095.58 | 833.59 | 1869.49 | 9.16 |
| 5 | 1291.55 | 7018.93 | 160.96 | 45.47 | 8553.17 | 102236.03 | 13279.45 | 4008.91 | N/A |
| 6 | 2277.53 | 9409.9 | 110.83 | 84.58 | 332.81 | 8640.05 | 215.00 | 1751.42 | 6.86 |
| 7 | 2124.98 | 9049.51 | 155.17 | 321.62 | 904.67 | 11986.60 | 558.78 | 2145.37 | N/A |
| 8 | 903.27 | 6894.13 | 105.14 | 87.04 | 1284.07 | 48390.04 | 824.74 | 1772.93 | N/A |
| Control group | |  |  |  |  |  |  |  |  |
| 1 | 2698.13 | 10232.13 | 117.82 | 23.46 | 521.16 | 3139.68 | 329.35 | 2741.78 | 10.24 |
| 2 | 1323.11 | 6877.52 | 64.4 | 26.54 | 702.49 | 3894.054 | 461.69 | 453.69 | N/A |
| 3 | 3995.89 | 12085.48 | 114.86 | 74.53 | 417.11 | 3482.94 | 232.23 | 1222.23 | N/A |
| 4 | 912.23 | 5267.19 | 61.72 | 17.65 | 1209.98 | 11937.04 | 773.27 | 928.76 | 18.35 |
| 5 | 1220.00 | 6226.59 | 47.87 | 13.56 | 312.47 | 5493.052 | 318.60 | 607.41 | N/A |
| 6 | 2044.3 | 8458.93 | 91.8 | 31.04 | 1000.63 | 5300.77 | 763.83 | 2574.93 | 20.21 |
| 7 | 1765.35 | 8169.53 | 96.52 | 62.81 | 696.25 | 13437.29 | 539.48 | 781.80 | N/A |
| 8 | 530.85 | 3150.4 | 65.58 | 4.85 | 17363.54 | 12465.06 | 18821.54 | 1098.50 | N/A |
| 9 | 2652.13 | 10547.67 | 110/00 | 30.86 | 1409.76 | 7382.70 | 590.529 | 311.81 | N/A |
| 10 | 1712 | 9343.30 | 106.84 | 31.21 | 991.72 | 6515.86 | 578.62 | 682.22 | N/A |
| 11 | 1248.12 | 6288.12 | 109 | 7.57 | 6063.60 | 14325.12 | 3522.09 | 991.09 | 46.58 |
| 12 | 4317.82 | 13519.8 | 163.43 | 77.87 | 428.88 | 5213.74 | 488.99 | 1075.88 | N/A |
| 13 | 2401.22 | 8323.71 | 91.11 | 16.37 | 738.24 | 8862.98 | 682.84 | 667.37 | N/A |
| 14 | 2158.84 | 9502.78 | 114.04 | 47.84 | 659.72 | 850.27 | 527.09 | 1097.88 | 9.92 |
| 15 | 2150.29 | 8938.97 | 84.03 | 8.60 | 365.95 | 6788.72 | 223.13 | 504.17 | 6.35 |
| 16 | 3946.12 | 13827.85 | 112.37 | 31.37 | 638.342 | 7158.44 | 387.56 | 1491.26 | N/A |
| 17 | 2742.44 | 10621.38 | 129.64 | 24.98 | 1793.61 | 11129.63 | 2476.51 | 1065.49 | N/A |
| 18 | 2521.40 | 10687.80 | 130.75 | 86.48 | 394.62 | 5422.38 | 198.065 | 514.44 | N/A |

*: below the detection limit.

N/A: not available.

**Supplementary Table S3:** Regression analyses of clinical status and biomarkers.

|  | Lowest score of GCS | | | | |
| --- | --- | --- | --- | --- | --- |
|  | Univariate analyses | | Multivariate analyses (age-adjusted) | | |
|  | ρ: | P: | B ± SE | | P: |
| CSF Aβ_1-42_ | 0.131 | 0.618 |  | |  |
| CSF Aβ_1-40_ | 0.063 | 0.811 |  | |  |
| CSF Aβ_1-42_ /Aβ_1-40_ | 0.190 | 0.465 |  | |  |
| CSF t-tau | -0.814 | <0.001** | -0.168x10^-3^±0.0910x10^-3^ | | 0.065 |
| CSF p-tau | -0.274 | 0.287 |  | |  |
| CSF p-tau /t-tau | 0.257 | 0.319 |  | |  |
| CSF NfL | -0.583 | 0.014* | -0.325x10^-3^±0.132x10^-3^ | | 0.014* |
| CSF p-NfH | -0.534* | 0.027* | -0.162x10^-3^±0.0900x10^-3^ | | 0.071 |
| CSF sTREM2 | -0.332 | 0.194 |  | |  |
| CSF GFAP | -0.443 | 0.075 |  | |  |
| Serum NfL | -0.804 | 0.007** | -0.48±0.022 | | 0.030* |
|  | mRS at discharge | | | | |
|  | Univariate analyses | | Multivariate analyses (age-adjusted) | | |
|  | ρ: | P: | B ± SE | | P: |
| CSF Aβ_1-42_ | -0.508 | 0.037* | -2.051x10-3±1.436x10-3 | | 0.161 |
| CSF Aβ_1-40_ | -0.334 | 0.189 |  | |  |
| CSF Aβ_1-42_ /Aβ_1-40_ | -0.488 | 0.047* | -20.539±13.9 | | 0.142 |
| CSF t-tau | 0.592* | 0.012* | -0.173x10^-3^±0.0966x10^-3^ | | 0.073 |
| CSF p-tau | -0.303 | 0.237 |  | |  |
| CSF p-tau /t-tau | -0.652 | 0.004** | -1.273± 0.708 | | 0.072 |
| CSF NfL | 0.683 | 0.002** | 0.373x10^-3^±0.158x10^-3^ | | 0.018* |
| CSF p-NfH | 0.691 | 0.002** | 0.014x10^-3^±0.009x10^-3^ | | 0.129 |
| CSF sTREM2 | 0.163 | 0.531 |  |  |  |
| CSF GFAP | 0.404 | 0.108 |  |  |  |
| Serum NfL | 0.812 | 0.007** | 0.065±0.041 | | 0.108 |

Results of uni- and multivariate regression analyses of the biomarkers and GCS/mRS are shown. “*”: P<0.05. “**”:P<0.01

**Supplementary Figure 1**

Comparison of biomarker concentrations among the HSV, VZV, and control groups ( A: CSF Aβ_1-42_, B: CSF Aβ_1-40_, C: CSF Aβ_1-42/1-40_ ratio, D: CSF t-tau, E:CSF p-tau, F: CSF p-tau/t-tau ratio, G: CSF NfL, H: CSF pNfH, I: CSF sTREM2, J: CSF GFAP, and K: serum NfL).

The CSF (HSV group: n=9, VZV group: n=8, control group: n=18) and serum (HSV group: n=6, VZV group: n=3, control group: n=6) levels of those biomarkers in each individual are shown as a black circle. Bars indicate median values. When multiple comparisons failed to reach significance, “n.s.” (not significant) was placed on n-shaped zig-zag lines. When a significant difference was observed among the groups, P-values in the subsequent post-hoc analyses were placed on the simple lines. (Unless otherwise specified, P-values on multiple comparisons were omitted.)

There were four significant differences on multiple comparison among the three groups: First, the levels of CSF t-tau were significantly higher in the HSV than control group (P = 0.0016, Suppl Fig1D). Second, CSF p-tau levels were significantly elevated in the VZV group (P=0.0102) and HSV (P=0.0487) groups compared with the control group (Suppl Fig 1E). Third, the CSF p-tau/t-tau ratio was significantly higher in the VZV compared not only with the control but also the HSV group (P=0.0389 and P=0.0291, respectively, Suppl Fig 1F). Fourth, levels of CSF GFAP were significantly increased in the HSV compared with the control group (P=0.0202, Suppl Fig 1J). The HSV and VZV groups trended to have higher CSF sTREM2 and lower CSF Aβ_1-42_ levels compared with the control. However, these trends did not reach significance (Suppl Fig 1I and 1A).

**
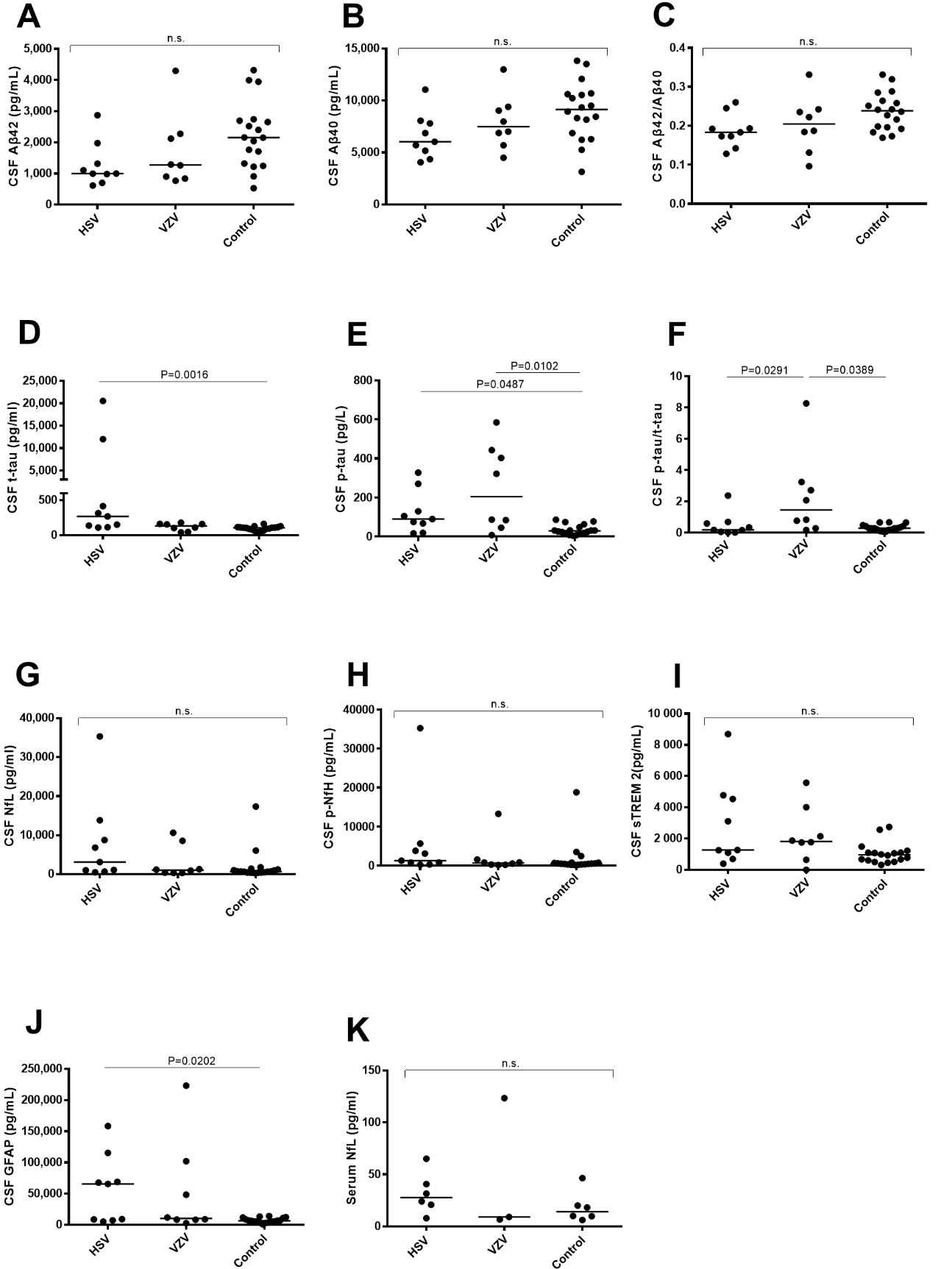
**

**Supplementary Figure 2**

Scatter plots of the biomarkers vs. the lowest score of GCS during the hospitalization (A, B, C, D) as well as the biomarkers vs. mRS score at discharge (E, F, G, H, I, J, K) in the HSV/VZV group. Note: graphs only shown when there was significant correlation on univariate correlation analysis. A: CSF t-tau vs. GCS, B: CSF NfL vs. GCS, C: CSF pNfH vs. GCS, D: serum NfL vs. GCS, E: CSF Aβ_1-42_ vs. mRS, F: CSF Aβ_1-42/1-40_ ratio vs. mRS, G: CSF t-tau vs. mRS, H: CSF p-tau/t-tau ratio vs. mRS, I: CSF NfL vs. mRS, J: CSF pNfH vs. mRS, K: serum NfL vs. mRS). Y-axes of graphs show concentrations of biomarkers using a logarithmic scale. X-axes represent GCS or mRS scores.

Univariate analyses showed significant negative correlations between the lowest score of GCS during the hospitalization and levels of CSF t-tau (Supple Fig 2A), CSF NfL (Suppl Fig 2B), CSF p-NfH (Suppl Fig 2C), and serum NfL (Suppl Fig 2D) (P< 0.001, P=0.014, P=0.027, and P=0.007 respectively). The levels of CSF Aβ _1-42_ (Suppl Fig 2E), CSF Aβ _1-42/1-40_ ratio (Supple Fig 2F), CSF t-tau (Suppl Fig 2G), CSF p-tau/t-tau ratio (Suppl Fig 2H), CSF NfL (Suppl Fig 2I), CSF p-NfH (Suppl Fig 2J), and serum NfL (Suppl Fig 2K) were significantly correlated with mRS on discharge (P= 0.037, P=0.047, P=0.012, P= 0.004, P=0.002, P=0.002, and P=0.007, respectively). The correlation coefficient of the association between CSF t-tau and mRS on discharge was positive (i.e., high t-tau levels associated with poor prognosis). Consequently, the correlation coefficient between the CSF p-tau/t-tau ratio (equivalent to reciprocal of CSF t-tau) and mRS on discharge was negative. CSF NfL, CSF pNfH and serum NfL were also positively associated with a poor prognosis. On the other hand, CSF Aβ _1-42_ and the CSF Aβ _1-42/1-40_ ratio were negatively correlated with the mRS scores (i.e., low Aβ _1-42_ levels and Aβ _1-42/1-40_ ratio associated with poor prognosis).

**
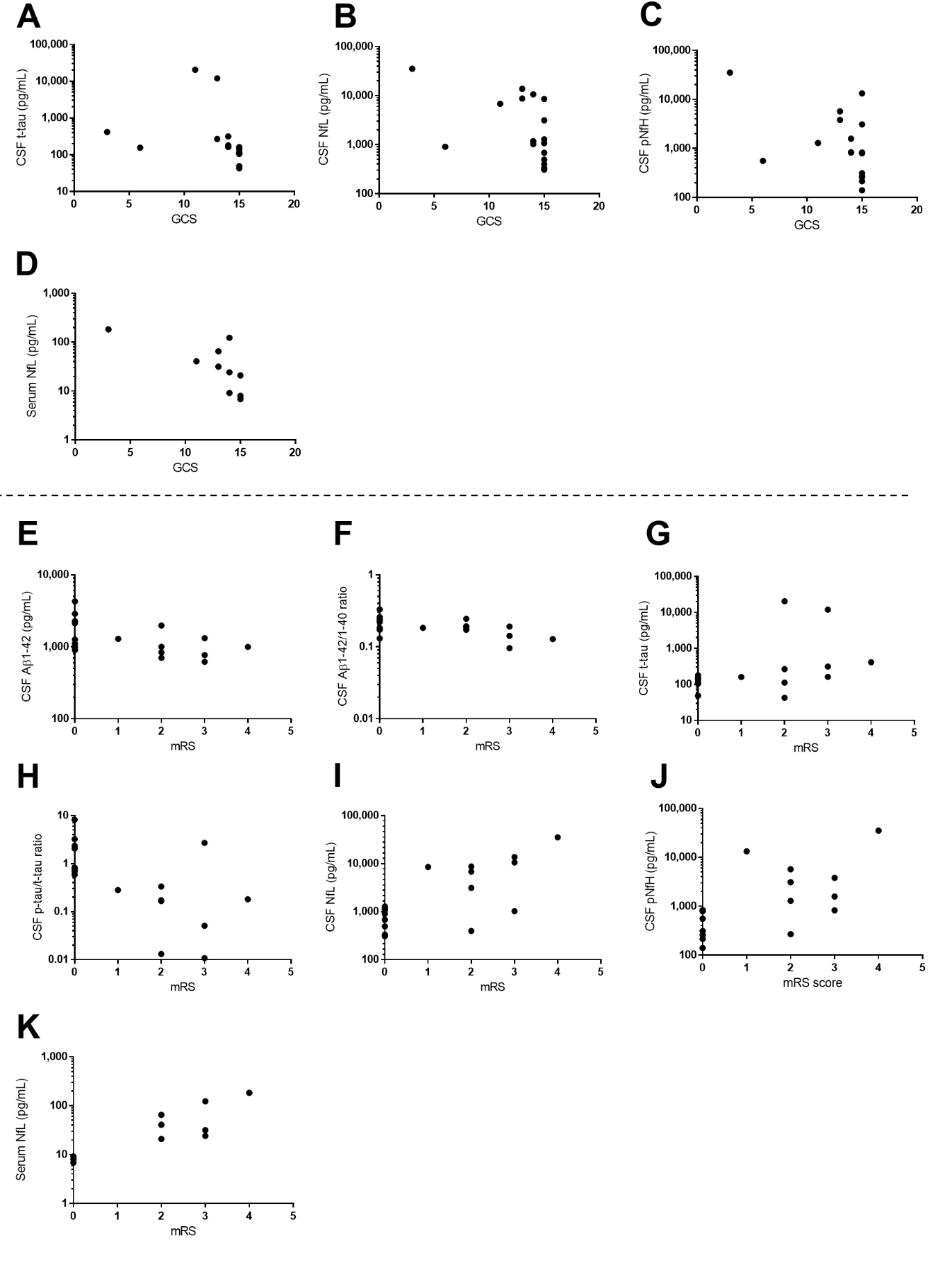
**

**Supplementary Figure 3**

Correlations between CSF and serum NfL levels. There was a strong positive correlation between CSF and serum NfL levels by Spearman’s rank correlation test (*p*<0.0001).
